# Microwave Foreign Object Detection in a Lossy Medium Using a Planar Array Antenna

**DOI:** 10.3390/s25133965

**Published:** 2025-06-26

**Authors:** Longzheng Yu, Peng Xu, Wenbo Li, Xiao Cai

**Affiliations:** 1 Research Center of Applied Electromagnetics, Nanjing University of Information Science and Technology, Nanjing 210044, China; yulongzheng0809@126.com (L.Y.); 202483270258@nuist.edu.cn (W.L.); 2 School of Information Engineering, Jiangsu Maritime Institute, Nanjing 211100, China; xup2016@126.com

**Keywords:** foreign object detection, lossy medium, method of the maximum power transmission efficiency, microstrip patch array antenna, non-contact detection

## Abstract

The non-contact detection of foreign objects embedded in lossy dielectric media such as soil, vegetation, or ice remains a critical challenge in applications including environmental monitoring and agricultural safety. This communication presents the design and experimental validation of an array antenna system capable of accurately localizing foreign objects in such lossy mediums. The proposed array antenna is capable of focusing electromagnetic energy at the location of the foreign object, thereby enabling precise positioning. The main idea of the foreign object detection is to set some of the antenna elements as test receiving antennas and measure the scattering parameters between the transmitting antennas and the receiving antennas. The excitation distribution of the transmitting array is optimized by using the method of maximum power transmission efficiency based on the differential scattering parameter matrices with the absence and presence of the foreign object. To validate the proposed design, a 5 × 5 microstrip patch array antenna was fabricated and tested with colza oil as a lossy medium. A copper block immersed in the colza oil served as the foreign object for detection, demonstrating the feasibility of the non-contact detection scheme. Experimental results demonstrate that the radiated field can be effectively focused at the object location, confirming the feasibility and precision of the proposed non-contact detection approach.

## 1. Introduction

Microwave antenna arrays have become indispensable in modern microwave inspection systems, enabling the non-contact sensing and detection of obscured targets embedded in diverse lossy mediums, including agricultural soils, glacier layers, and coastal sediments. Among various types, focused antennas, a type of antenna that can concentrate electromagnetic wave energy, are particularly attractive for their ability to concentrate electromagnetic energy into a specific region, thereby enhancing signal transmission efficiency and improving reception sensitivity. Focused antenna systems have been widely applied in areas such as near-field communications [1,2,3,4,5,6,7], target imaging [8,9,10], microwave hyperthermia [11,12,13,14,15,16], wireless power transfer [17,18,19,20,21,22], and microwave industrial inspections [23,24,25]. In the microwave frequency band, electric field focusing can be achieved by lens antennas [26,27], reflector antennas [28] and array antennas [29], among which focusing array antennas offer superior flexibility by enabling energy concentration at arbitrary locations through tailored excitation distributions. Several design methodologies have been developed to achieve the desired focusing performance, including quasi-optical methods [30], time-reversal [31], global optimization [13], and the method of maximum power transmission efficiency (MMPTE) [32,33,34].

Traditional focusing array antennas are typically designed to concentrate the electric field at one or more predefined locations. To determine the excitation weights of such arrays, it is crucial to first obtain the electromagnetic characteristics of the intended focal region and its surrounding environment. For example, in microwave hyperthermia, focused array antennas rely on the precise knowledge of the dielectric properties of biological tissues and the exact position of the tumor [14,15]. This information is commonly acquired through advanced medical imaging techniques, such as magnetic resonance imaging or computed tomography. Moreover, in some cases, a receiving antenna must be inserted into the lesion area to facilitate the calculation of the appropriate excitation distribution. However, these methods face significant limitations when the electromagnetic properties of the environment are unknown or cannot be accurately characterized. In such scenarios, conventional focusing techniques, which depend heavily on the prior knowledge of the target and its surroundings, become impractical. This challenge underscores the need for alternative strategies capable of achieving accurate field focusing without relying on predefined environmental information.

This communication proposes a novel non-contact foreign object detection method for identifying foreign objects in lossy media with unknown characteristics. Based on the scattering parameter matrix obtained from the system’s scattering parameters, the proposed method enables the electromagnetic field to be focused at arbitrary target positions—without requiring prior knowledge of the target’s location, shape, or dielectric properties. To validate the proposed approach, a 5 × 5 microstrip patch array antenna was designed, fabricated, and tested. Experimental results demonstrate that the array antenna can accurately focus the field onto copper block targets randomly distributed within a lossy medium, thereby achieving effective and precise non-contact detection.

## 2. Method of Maximum Power Transmission Efficiency

Based on the MMPTE reported in [20], the transmitting array antenna and receiving antenna can be considered as a (24 + 1)-port network. The focusing effect in the target object area can be achieved by maximizing the power transmission efficiency η from the transmitting array antenna to the receiving antenna. This system can be represented by the following scattering parameter matrix:

(1) $$\left[\begin{array}{c} \left[b_{t}\right]\\ \left[b_{r}\right] \end{array}\right]=\left[\begin{array}{cc} \left[S_{tt}\right] & \left[S_{tr}\right]\\ \left[S_{rt}\right] & \left[S_{rr}\right] \end{array}\right]\left[\begin{array}{c} \left[a_{t}\right]\\ \left[a_{r}\right] \end{array}\right]$$

where the subscript “*t*” represents the transmitting array, and the subscript “*r*” represents the receiving antenna. $[a_{t}]={\left[a_{1},a_{2},\cdots ,a_{n}\right]}^{T}$, $[b_{t}]={\left[b_{1},b_{2},\cdots ,b_{n}\right]}^{T}$ represent the normalized incident wave and reflected wave of the transmitting antenna port, respectively, and $\left[a_{r}]=[a_{n+1}\right]$, $\left[b_{r}]=[b_{n+1}\right]$ represent the normalized incident wave and reflected wave of the receiving antenna port, respectively (*n* = 24). The transmission efficiency between the transmitting array antenna and the receiving antenna can be represented by the ratio $T_{array}$ between the power received by the receiving antenna and the output power of the transmitting array.

(2) $$T_{array}=\frac{\frac{1}{2}(|[b_{r}]{|}^{2}-|[a_{r}]{|}^{2})}{\frac{1}{2}(|[a_{t}]{|}^{2}-|[b_{t}]{|}^{2})}.$$

When the receiving antenna is perfectly matched, i.e., $[a_{r}]=0$, the formula can be simplified as follows:

(3) $$T_{array}=\frac{\left([A][a_{t}],[a_{t}]\right)}{\left([B][a_{t}],[a_{t}]\right)}.$$

When the system is fully matched, if $T_{array}$ reaches its maximum, the following eigenvalue equation can be obtained:

(4) $$[A][a_{t}]=T_{array}[a_{t}].$$

In this letter, matrix [*A*] is derived from the difference value in the scattering parameter matrix between the receiving antenna and the transmitting antenna in the absence and presence of a foreign object. The scattering parameter matrix between the receiving antenna and the transmitting antenna in the absence of a foreign object is $\left[S_{rt1}\right]$, and the scattering parameter matrix between the receiving antenna and the transmitting antenna in the presence of a foreign object is $\left[S_{rt2}\right]$. Thus, the difference value $\Delta [S_{rt}]$ between the two scattering parameter matrices can be calculated by

(5) $$\Delta [S_{rt}]=[S_{rt2}]-[S_{rt1}].$$

Then, the new matrix [*A*] is calculated by

(6) $$\left[A]=\Delta {\left[S_{rt}\right]}^{H}\Delta [S_{rt}\right]$$

where the superscript ‘*H*’ represents the Hermitian operation.

The subtraction of scattering parameter matrices effectively eliminates most background interference, thereby enhancing the robustness of the proposed non-contact detection method against environmental noise and enabling high-resolution detection. The maximum power transmission efficiency between the transmitting and receiving antennas corresponds to the unique non-zero eigenvalue of (4), and its associated eigenvector represents the optimal excitation distribution for the array antenna. By applying this excitation in the absence of the target and simulating the resulting electric field distribution within the lossy medium, the target location can be identified as the focal point of the field. This enables the precise non-contact detection of foreign objects without requiring invasive measurements.

Furthermore, when the target position changes, only the updated scattering parameter matrix needs to be measured. The difference between the new and baseline scattering parameters can then be used to recalculate the optimal excitation, allowing the system to dynamically track the target’s location. This feature indicates that the proposed method has strong adaptability and accuracy in real-time and evolving detection scenarios.

## 3. Array Antenna Design

**Figure 1:**
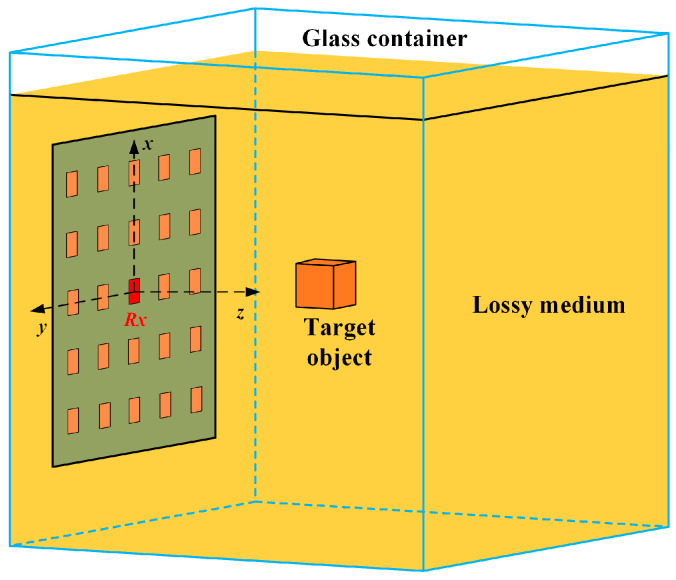
Diagram of the proposed non-contact detection array antenna system.

To validate the proposed non-contact detection scheme for foreign objects in the lossy medium, a 5 × 5 rectangular microstrip patch antenna array was designed, fabricated and tested. The array antenna exhibits symmetry in the horizontal direction (*y*-axis) but slight asymmetry in the vertical direction (*x*-axis). The detection operates within a lossy medium composed of colza oil, which emulates typical dielectric properties found in practical applications. As illustrated in Figure 1, the array antenna is mounted outside a glass container filled with the oil medium, and a copper block, serving as the foreign object, is suspended within the oil at various positions for detection.

**Figure 2:**
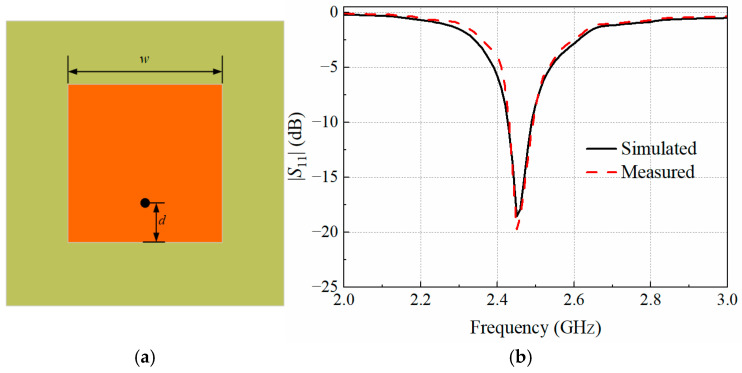
(**a**) Diagram of patch element; (**b**) the simulated and measured reflection coefficient of the patch element.

The array elements are implemented as rectangular microstrip patch antennas operating at 2.45 GHz. The experimental environment consists of a glass container filled with colza oil, which has a relative dielectric constant of 2.2 and a loss tangent of 0.00079. The dimensions of the glass container are 35 × 35 × 40 cm^3^ with a thickness of 5 mm, while the volume of the colza oil is about 35 × 35 × 35 cm^3^. Each patch element was fabricated on a 1.6 mm thick FR4 substrate, which has a relative dielectric constant of 4.4 and a loss tangent of 0.02. The geometric parameters of the antenna element were optimized using Ansoft HFSS software (version 16.1.0), yielding optimal dimensions for the rectangular patch element of *w* = 25.2 mm and *d* = 7 mm. Figure 2a and Figure 2b show the schematic diagram of the patch element along with its simulated and measured reflection coefficients, respectively. Based on the optimized patch element, a 5 × 5 array antenna is configured, where the central element serves as the test receiving antenna while the remaining elements form the transmitting array antenna, enabling the proposed non-contact detection of foreign objects.

## 4. Results and Discussion

The center position of the array antenna is designated as the origin of the rectangular coordinate system. A copper block (a cube with a side length of 20 mm) is placed at four distinct positions within the near-field region: P1 (0, 0, 60 mm), P2 (60, 0, 110 mm), P3 (−60, 0, 110 mm), and P4 (60, 60, 110 mm). Based on the proposed non-contact detection scheme, the optimal distribution of excitations (ODE) for the transmitting array can be determined without prior knowledge of the target’s size, shape, or electromagnetic properties. When the electromagnetic waves radiated by the transmitting array interact with the target, the resulting scattered field is captured by the receiving antenna. The scattering parameters between the transmitting and receiving elements are measured using a vector network analyzer (VNA). Two sets of *S*-parameter data are acquired: one with the foreign object present and the other without it. By calculating the difference between these two datasets, the excitation distribution of the transmitting array is optimized using the MMPTE to focus the electric field at the target location. The measured scattering parameters under both conditions are presented in Table 1 and Table 2.

The electric field distribution within the lossy medium is simulated under the condition without the target object. The location exhibiting the highest electric field intensity corresponds to the target’s position. When the target position changes, the scattering parameters for the new position are remeasured. By applying the updated difference in scattering parameters to solve Equation (4), a new ODE is derived to refocus the electric field on the target’s new position. Through iterative calculations of the electric field distribution, the non-contact detection of the target’s location is achieved.

The proposed method requires only two measurements: one in the absence of the foreign object and another in its presence. Based on the differential scattering parameters, the excitation distribution of the transmitting array is calculated, and the target’s position is determined by analyzing the resulting electric field distribution. Notably, this approach does not depend on any prior knowledge of the target’s size, shape, or electromagnetic properties, making it highly versatile and suitable for a wide range of application scenarios.

ODEs for non-contact detection at these four positions are listed in Table 3. These ODEs are realized by using a radio frequency feeding circuit, which includes power dividers, phase shifters, and attenuators. The power dividers provide 24 output channels to generate the appropriate excitation for the transmitting array. Attenuators and phase shifters are employed to adjust the amplitude and phase of the output excitation, respectively. Additionally, the feeding circuit board incorporates a boost circuit to stabilize the voltage and enhance the adjustable output voltage amplitude.

**Figure 3:**
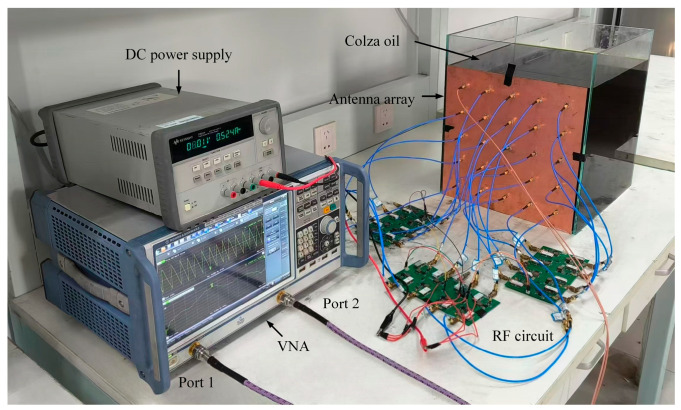
Photograph of the proposed non-contact detection array antenna system.

The complete experimental setup is illustrated in Figure 3. A VNA is used to measure the scattering parameters, and a DC power supply energizes the feeding circuit. This setup enables the accurate excitation control and measurement, thereby validating the effectiveness of the proposed non-contact detection method.

**Figure 4:**
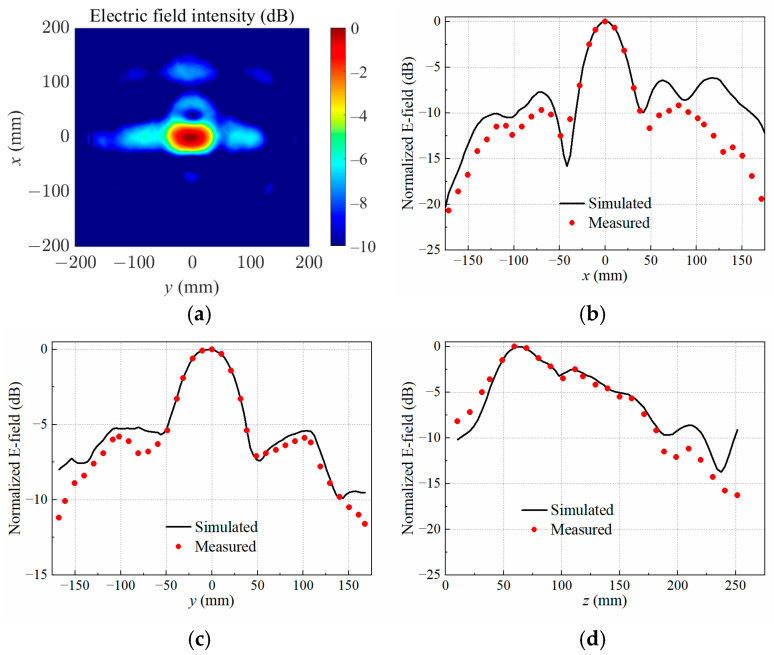
Simulated electric field distribution (**a**) in the target focal plane at *z* = 60 mm, and simulated and measured results along different axes crossing the target center at P1: (**b**) *x*-axis; (**c**) *y*-axis; (**d**) *z*-axis.

The data of electric field intensity as well as electric field distributions were obtained through full-wave simulations in HFSS. When the copper block was positioned at P1, the simulated normalized electrical field distribution at the target focal plane was *z* = 60 mm, as illustrated in Figure 4a. Furthermore, Figure 4b–d present a comprehensive comparison between the measured and simulated normalized electric field distributions along the *x*-, *y*-, and *z*-axes across the center of the copper block. The experimental data were acquired using a precise electric field probe in conjunction with a VNA, ensuring high accuracy and reliability. The spatial resolution of the measurements was maintained at a step size of 10 mm, allowing for a detailed and consistent mapping of the electric field intensity.

The results demonstrate an agreement between the measured and simulated electric field distributions, both in the focal *xy*-plane and along the *z*-axis. This consistency underscores the validity of the simulation model and the precision of the experimental setup. Minor discrepancies, if any, can be attributed to practical limitations such as probe alignment, environmental noise, or slight variations in the material properties of the copper block.

**Figure 5:**
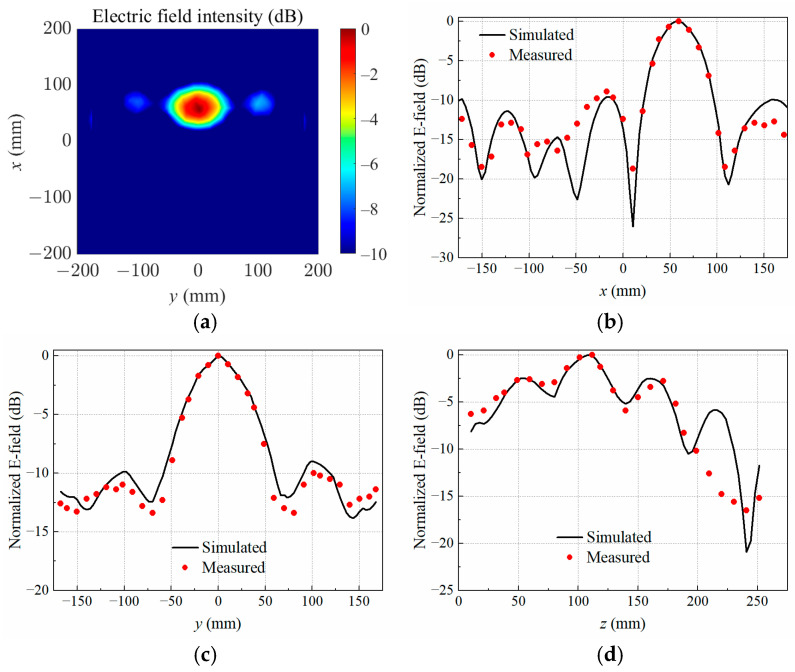
Simulated electric field distribution (**a**) in the target focal plane at *z* = 110 mm, and simulated and measured results along different axes crossing the target center at P2: (**b**) *x*-axis; (**c**) *y*-axis; (**d**) *z*-axis.

**Figure 6:**
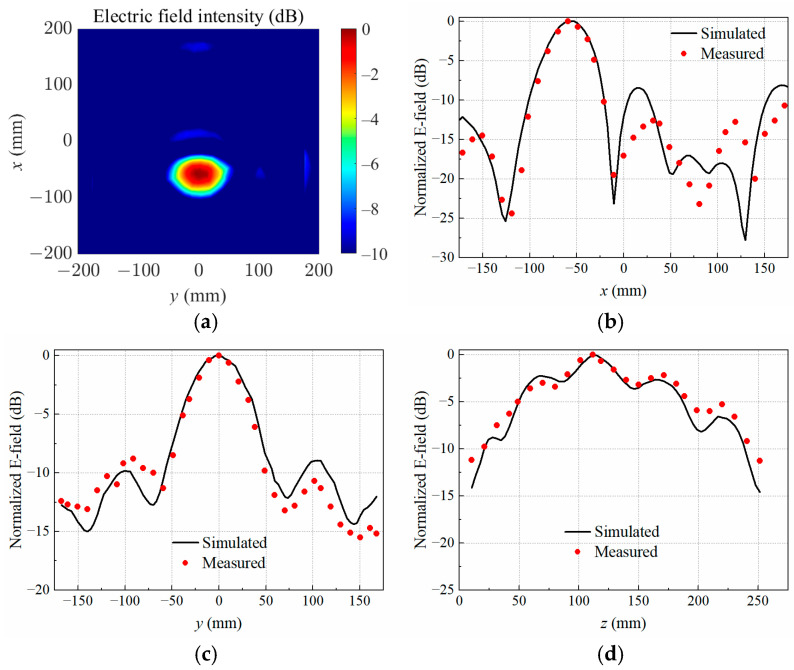
Simulated electric field distribution (**a**) in the target focal plane at *z* = 110 mm, and simulated and measured results along different axes crossing the target center at P3: (**b**) *x*-axis; (**c**) *y*-axis; (**d**) *z*-axis.

**Figure 7:**
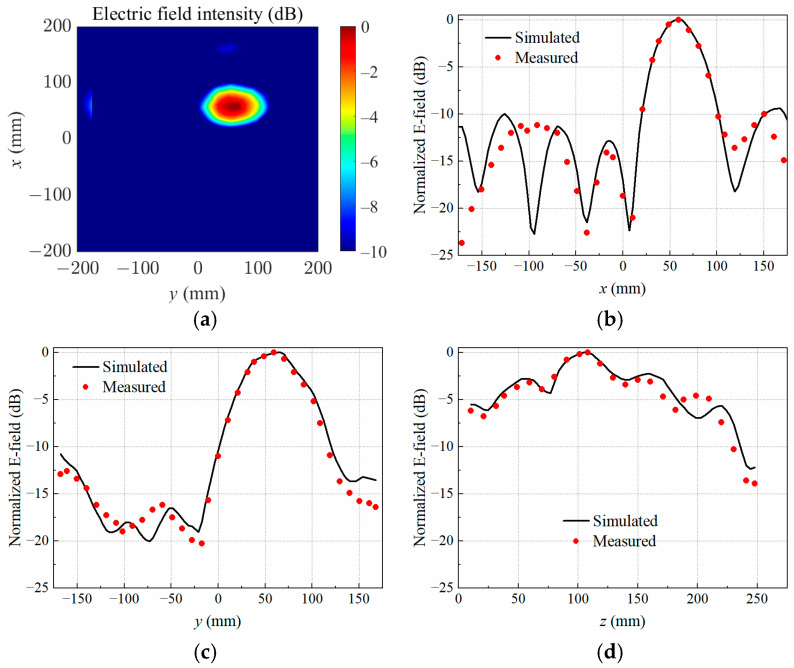
Simulated electric field distribution (**a**) in the target focal plane at *z* = 110 mm, and simulated and measured results along different axes crossing the target center at P4: (**b**) *x*-axis; (**c**) *y*-axis; (**d**) *z*-axis.

When the copper block was placed at P2, P3, and P4, the simulated normalized electrical field distributions at the target focal plane *z* = 110 mm are shown in Figure 5a, Figure 6a and Figure 7a, respectively. The measured and simulated electric field distributions across the target center along the *x*-, *y*-, and *z*-axes are shown in Figure 5b–d, Figure 6b–d, and Figure 7b–d. It was observed that the transmitting array antenna was able to focus the electric field on the position of the copper block as expected, achieving the non-contact detection of a foreign object in lossy medium.

To comprehensively evaluate the non-contact detection range of the system, the copper block was positioned at various locations along the *x*-axis: P5 (80, 0, 80 mm), P6 (100, 0, 80 mm), P7 (120, 0, 80 mm), and P8 (130, 0, 80 mm).

**Figure 8:**
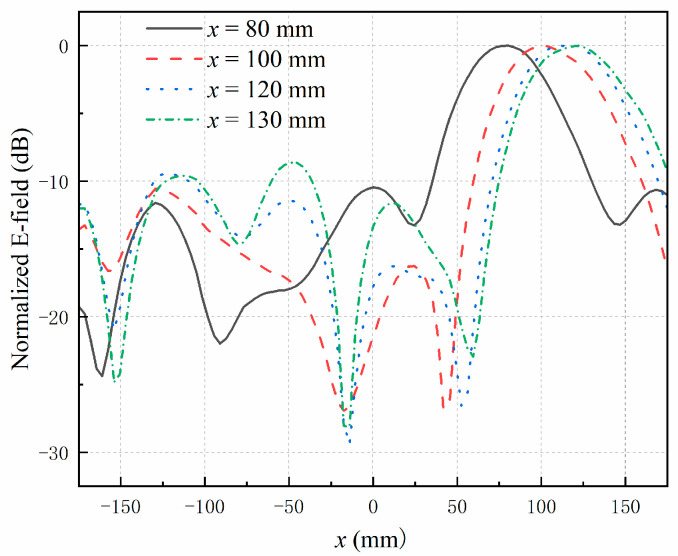
Simulated electric field distributions along *x*-axis when the target is located at different positions.

The simulated electric field distributions along the *x*-axis are shown in Figure 8. The results indicate that the maximum effective detection range of the array antenna is approximately 120 mm, corresponding to the distance from the outermost patch element to the center of the array. Within this range, the array exhibits a strong capability to focus the electric field and accurately detect the presence of the copper block. This limitation arises because the differential scattering parameters become too weak to distinguish the presence of the foreign object reliably. As the distance increases, the received signal strength diminishes, reducing the sensitivity of the system.

In practical applications, the conductivity and permittivity of the target, as well as the dielectric values and loss tangent of the surrounding medium, will inevitably affect the detection performance. Thus, a comparison is provided in Table 4 for different targets in the same lossy medium.

**Figure 9:**
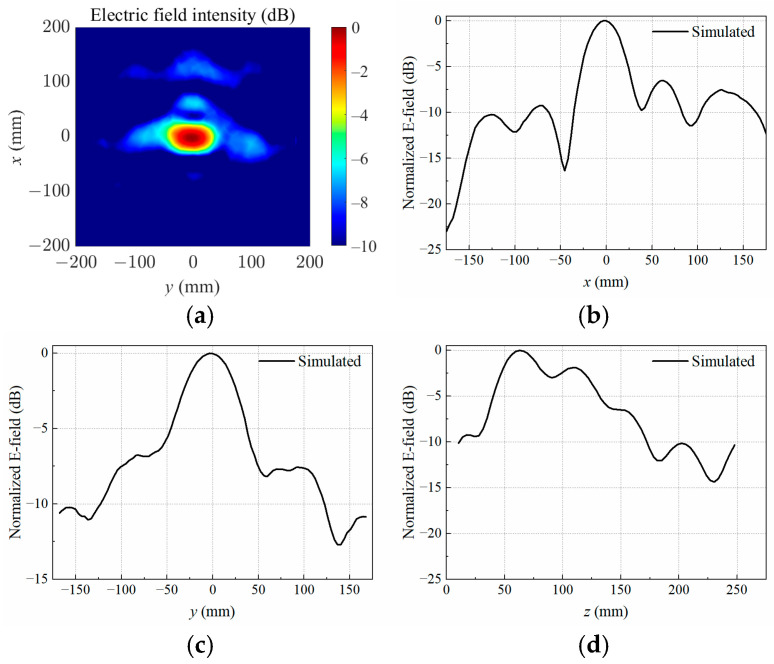
Simulated electric field distribution when the non-metallic target is located at P1 (**a**) in the target focal plane at *z* = 60 mm; (**b**) *x*-axis; (**c**) *y*-axis; (**d**) *z*-axis.

**Figure 10:**
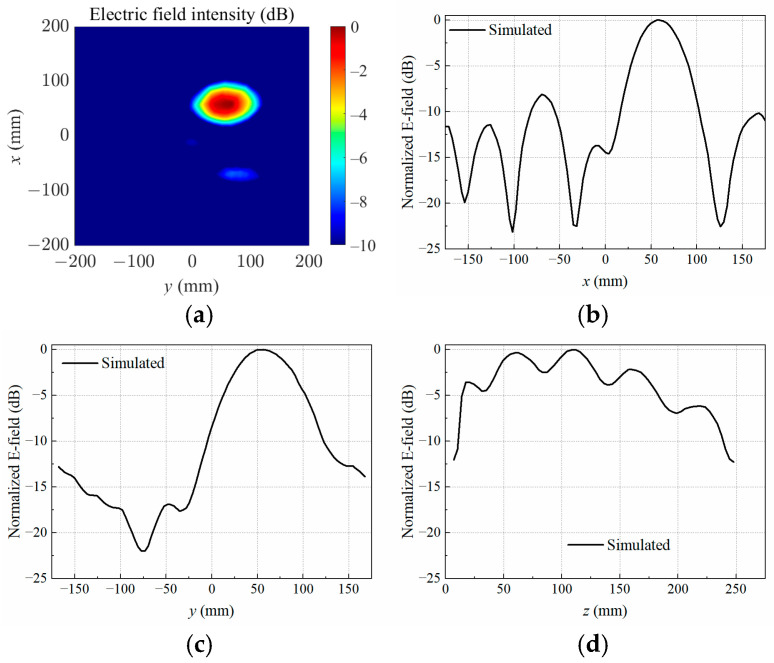
Simulated electric field distribution when the non-metallic target is located at P4 (**a**) in the target focal plane at *z* = 110 mm; (**b**) *x*-axis; (**c**) *y*-axis; (**d**) *z*-axis.

The simulated results show that the system demonstrates effective detection capabilities for non-metallic targets. As an example, a square non-metallic target with a side length of 40 mm and thickness of 3 mm was selected as the foreign target, with colza oil serving as the lossy medium. The target was placed at two positions that were the same as the copper block: P1 (0, 0, 60 mm) and P4 (60, 60, 110 mm). The simulated results shown in Figure 9 and Figure 10 confirm that although the target has a low conductivity compared to the copper block, the array antenna maintained a good focusing effect for accurate detection.

As clearly demonstrated in Figure 9 and Figure 10, the focal point precisely aligns with the target’s actual position, confirming the system’s effective detection capability. These results robustly validate that the presented system can accurately detect targets exhibiting both relatively low conductivity and relatively high dielectric values.

The target positioning performance of the system was also tested in different media with different loss tangents. Additional simulations were carried out with an unchanged dielectric constant and an increasing loss tangent, while the targets were set at the position of (0, 0, 85 mm).

**Figure 11:**
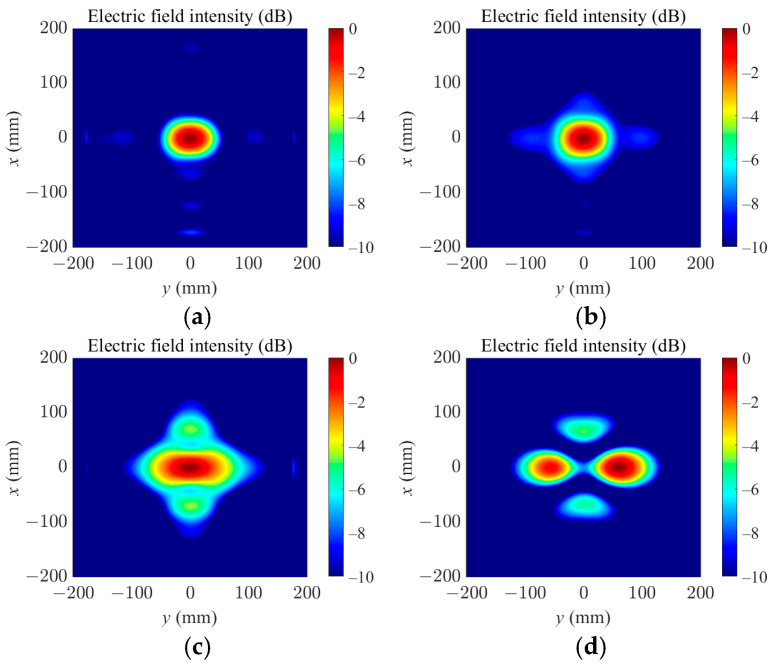
Simulated electric field distribution in the target focal plane at *z* = 85 mm when the loss tangent of the medium is set as (**a**) 0.05; (**b**) 0.1; (**c**) 0.2; (**d**) 0.3.

Figure 11 shows the simulated results in the target focal plane *z* = 85 mm when the loss tangent of the medium was set as 0.05, 0.1, 0.2, and 0.3. For a small loss tangent, such as the colza oil with a loss tangent of 0.00079 used in this paper, a good focusing effect is achieved by the proposed optimization process. It can be seen that when the loss tangent of the medium reaches 0.3, the focal point split into two points, losing the detection effect of the target, since it is difficult for the electromagnetic wave to penetrate the high-loss medium. In this case, the system can no longer effectively detect the target.

## 5. Conclusions

This paper presents a non-contact detection method for identifying foreign objects embedded in a lossy medium by analyzing the differential scattering parameters. The measured difference in scattering parameters is incorporated into the MMPTE framework to solve an eigenvalue problem, yielding the ODE for the transmitting array. This excitation distribution enables the array to focus electromagnetic energy precisely at the location of foreign objects within the lossy medium, achieving effective non-contact detection. The proposed method does not require any prior knowledge of the object’s dielectric properties, geometry, and position. To validate the proposed method, a 5 × 5 microstrip patch array antenna was designed, fabricated, and experimentally tested using colza oil as the lossy medium. A copper block immersed in the oil served as the target object. Experimental results confirm that the transmitting array can accurately focus the electric field at the object’s location, and the simulated field distributions agree well with the measured data. These findings demonstrate the feasibility and accuracy of the proposed non-contact detection approach, highlighting its potential for non-contact sensing, microwave medical treatment, and microwave industrial inspections.

## Figures and Tables

**Table 1:** Scattering parameters of the array antenna when the target is absence and positioned at P1.

Antenna No.	Re (Absence)	Im (Absence)	Re (P1)	Im (P1)
1	–0.0263	0.0196	–0.0275	0.0344
2	0.0265	0.0074	0.0147	0.0077
3	0.0077	0.0316	–0.0090	0.0032
4	0.0060	–0.0164	–0.0107	–0.0141
5	–0.0005	0.0374	0.0086	0.0494
6	–0.0299	0.0029	–0.0468	0.0008
7	0.0019	–0.0272	0.0237	0.0019
8	–0.0042	–0.0282	–0.0383	0.0138
9	–0.0223	–0.0341	–0.0068	–0.0101
10	–0.0017	0.0288	–0.0089	0.0276
11	–0.0241	–0.0099	–0.0413	–0.0282
12	–0.0501	0.0453	–0.0594	0.0923
13	–0.0143	0.0320	–0.0273	0.0765
14	–0.0343	0.0053	–0.0412	–0.0058
15	–0.0230	0.0031	–0.0376	0.0037
16	0.0024	–0.0279	0.0216	–0.0066
17	–0.0125	–0.0143	–0.0371	0.0222
18	–0.0226	–0.0324	–0.0096	–0.0153
19	0.0042	0.0320	–0.0020	0.0311
20	–0.0250	0.0250	–0.0279	0.0359
21	0.0187	0.0099	0.0122	0.0119
22	0.0131	0.0250	–0.0035	0.0023
23	–0.0006	–0.0176	–0.0143	–0.0132
24	–0.0026	0.0404	0.0060	0.0495

**Table 2:** Scattering parameters of the array antenna when the target is positioned at P2, P3 and P4.

Antenna No.	Re (P2)	Im (P2)	Re (P3)	Im (P3)	Re (P4)	Im (P4)
1	–0.0262	0.0208	–0.0312	0.0153	–0.0288	0.0151
2	0.0221	0.0106	0.0294	0.0077	0.0278	–0.0015
3	0.0050	0.0294	0.0094	0.0342	0.0035	0.0275
4	0.0127	–0.0112	0.0031	–0.0133	0.0061	–0.0149
5	–0.0025	0.0347	0.0002	0.0394	0.0014	0.0363
6	–0.0216	–0.0014	–0.0314	–0.0021	–0.0266	0.0085
7	0.0145	–0.0200	0.0021	–0.0212	0.0058	–0.0152
8	0.0046	–0.0328	–0.0060	–0.0334	–0.0007	–0.0177
9	–0.0260	–0.0313	–0.0165	–0.0421	–0.0178	–0.0421
10	–0.0012	0.0266	–0.0041	0.0216	–0.0025	0.0335
11	–0.0162	–0.0068	–0.0192	–0.0138	–0.0380	–0.0178
12	–0.0524	0.0630	–0.0531	0.0492	–0.0522	0.0280
13	–0.0189	0.0327	–0.0074	0.0348	–0.0089	0.0403
14	–0.0284	0.0043	–0.0290	–0.0002	–0.0389	0.0028
15	–0.0162	–0.0010	–0.0250	–0.0020	–0.0267	–0.0141
16	0.0136	–0.0210	0.0033	–0.0229	0.0197	–0.0399
17	–0.0038	–0.0191	–0.0131	–0.0194	–0.0189	–0.0311
18	–0.0255	–0.0291	–0.0165	–0.0395	–0.0193	–0.0210
19	0.0037	0.0290	0.0017	0.0246	0.0026	0.0243
20	–0.0246	0.0254	–0.0296	0.0205	–0.0298	0.0219
21	0.0159	0.0120	0.0220	0.0099	0.0190	0.0021
22	0.0109	0.0237	0.0147	0.0282	0.0048	0.0200
23	0.0044	–0.0128	–0.0047	–0.0148	0.0070	–0.0105
24	–0.0042	0.0378	–0.0019	0.0417	–0.0050	0.0390

**Table 3:** ODEs of the array antenna for a target located at different positions.

Antenna No.	P1	P2	P3	P4
1	0.113∠−48°	0.034∠152.2°	0.227∠−158.3°	0.097∠−29.9°
2	0.090∠−132.2°	0.144∠95°	0.099∠55.8°	0.170∠−67.2°
3	0.250∠167.1°	0.092∠20.6°	0.109∠5.7°	0.110∠−13.1°
4	0.127∠−125.6°	0.223∠−158°	0.146∠−70.6°	0.029∠123.9°
5	0.114∠−5.8°	0.089∠5.4°	0.072∠−7.3°	0.042∠−117.9°
6	0.129∠−140.7°	0.247∠−92.7°	0.181∠168.8°	0.121∠152.6°
7	0.276∠−6.6°	0.383∠−150.3°	0.210∠−26.1°	0.236∠139.3°
8	0.409∠−82.5°	0.262∠−92.1°	0.194∠171.7°	0.207∠139.5°
9	0.217∠−10.7°	0.124∠97.2°	0.342∠116.8°	0.172∠−87.9°
10	0.055∠−143°	0.059∠−43.9°	0.263∠171.3°	0.089∠110.9°
11	0.190∠179.8°	0.223∠−141.8°	0.216∠100.9°	0.299∠1.6°
12	0.363∠−54.5°	0.469∠142.4°	0.169∠−64.5°	0.328∠−51.9°
13	0.352∠−59.7°	0.125∠68.4°	0.257∠40.5°	0.186∠154.2°
14	0.099∠168.7°	0.156∠−110.6°	0.266∠108.4°	0.099∠3.5°
15	0.110∠−131°	0.209∠−89.3°	0.190∠173°	0.330∠−46.9°
16	0.218∠−1.4°	0.348∠−151.7°	0.176∠−17.3°	0.395∠−114°
17	0.333∠−77.4°	0.262∠−91.3°	0.176∠159°	0.337∠−37.9°
18	0.163∠−6.1°	0.116∠108.7°	0.325∠159°	0.223∠137.6°
19	0.048∠−141.4°	0.081∠−21°	0.271∠111.5°	0.148∠−46.8°
20	0.086∠−58.5°	0.016∠−167.4°	0.225∠170.6°	0.108∠−1.9°
21	0.052∠−116.2°	0.095∠96.8°	0.113∠−161.9°	0.146∠−60.8°
22	0.213∠172.8°	0.068∠29.7°	0.125∠61°	0.181∠0.8°
23	0.110∠−115.7°	0.181∠−163.9°	0.172∠−1.4°	0.193∠168.2°
24	0.095∠0°	0.081∠0°	0.050∠−84.2°	0.053∠0°

**Table 4:** Comparison of the detection performance of different targets.

Target Material	Conductivity (S/m)	Dielectric Value	Medium	Detection	Maximum Electric Field Intensity (dB)
Copper	5.8 × 10^7^	1	Colza oil	Yes	30
Non-metallic	10^−14^	4.4	Colza oil	Yes	26

## Data Availability

Data are contained within the article.
